# Curcumin Mitigates Radiation-induced Lung Pneumonitis and Fibrosis in Rats

**DOI:** 10.22088/IJMCM.BUMS.7.4.212

**Published:** 2019-01-27

**Authors:** Paiman Amini, Hana Saffar, Mohammad Reza Nourani, Elahe Motevaseli, Masoud Najafi, Ramezan Ali Taheri, Ali Qazvini

**Affiliations:** 1 *Nanobiotechnology Research Centre, Baqiyatallah University of Medical Sciences, Tehran, Iran.*; 2 *Clinical and Anatomical Pathology, Tehran University of Medical Science, Imam Khomeini Hospital Complex, Tehran, Iran.*; 3 *Department of Molecular Medicine, School of Advanced Technologies in Medicine, Tehran University of Medical Sciences, Tehran, Iran.*; 4 *Radiology and Nuclear Medicine Department, School of Paramedical Sciences, Kermanshah University of Medical Sciences, Kermanshah, Iran.*; 5 *Department of Pulmonology, Faculty of Medicine, Baqiyatallah University of Medical Sciences, Tehran, Iran.*

**Keywords:** Curcumin, radiation, radioprotection, DUOX1, DUOX2

## Abstract

Radiation-induced lung injury is one of the most prominent factors that interfere with chest cancer radiotherapy, and poses a great threat to patients exposed to total body irradiation. Upregulation of pro-oxidant enzymes is one of the main mechanisms through which the late effects of ionizing radiation on lung injury can be exerted. Interleukin (IL)-4 and IL-13 are two important cytokines that have been proposed to be involved in this process. Through stimulation of dual oxidase 1 and 2 *(DUOX 1* & *2*), they induce chronic oxidative stress in irradiated tissues. In this study, we evaluated the effects of curcumin treatment on the regulation of *IL-4* and *IL-13*, *DUOX1* & *2 *genes as well as the pathological changes developed by this treatment. Twenty male Wistar rats were divided into four groups: radiation only; curcumin only; radiation +curcumin; and control group with neither pharmacotherapy nor radiation. Curcumin was administered for 4 and 6 consecutive days before and after irradiation, respectively. Also, the chest area was irradiated with 15 Gy using a cobalt-60 gamma rays source. All rats were sacrificed 67 days after irradiation, followed by the assessment of the levels of IL-4 and IL-13; the expression of IL- 4 receptor-a1 (*IL4Ra1*), *IL13Ra2*, *DUOX1* and *DUOX2*, and finally the histopathological changes were evaluated. Radiation led to the increased level of IL-4, while the level of IL-13 showed no change. QPCR results showed the upregulation of* IL4Ra1*, *DUOX1* and *DUOX2* following lung irradiation. Histopathological evaluation also showed a remarkable increase in pneumonitis and fibrosis. Treatment with curcumin downregulated the expression of *IL-4*, *IL4Ra1*, *DUOX1* & *2*. Furthermore, it could mitigate pneumonitis and fibrosis following lung irradiation. The late effects of radiation- induced lung injury may be due to the upregulation of *DUOX1* & *2* genes. Curcumin, through modulation of these genes, may contribute to the protection against ionizing radiation.

Tradiation-induced lung injury after radiotherapy or radiation accident is one of the most notable side effects of ionizing radiation. Exposure to an acute dose of radiation during a nuclear disaster or fractionated radiotherapy may interfere with normal function of the lung (1). Lung tissue responses to radiation includes pneumonitis and fibrosis, owing to excessive free radical production and chronic changes in immunological mediators (2). As these consequences may lead to the death of exposed persons, effective management of radiation-induced lung injury is of particular importance.

For many years, several studies have been conducted to evaluate the protective effects of different agents against toxic ionizing radiation. However, due to the considerable toxicity of these agents, there has been a growing interest towards natural and herbal agents (3). Amongst the various types of herbal agents, polyphenols such as curcumin are classified as the most important and well- known ones. Curcumin has been traditionally used to tackle inflammation and thrombosis as well as to prevent hepatic disorders (4). As a radio-protector, it has shown protective ability against radiation-induced oxidative stress, DNA damage, inflammation, fibrosis, etc.(5, 6). In a phase 1 clinical trial, administration of 8,000 mg/day curcumin as a chemopreventive agent showed no toxicity and contributed to the healing of patient's lesions (7).

To date, the mechanisms of radiation-induced inflammatory diseases and fibrosis are yet to be fully understood. However, long term upregulation of some cytokines and continuous free radical production lead to changes in the morphological structure of inter/ intracellular spaces. For example, chronic upregulation of transforming growth factor beta (TGF-β) stimulates reactive oxygen species (ROS) and nitric oxide (NO) production and collagen accumulation in intracellular space (8). So far, various signaling pathways have been proposed for TGF-β, among which TGF-β/Smad, TGF-β/Rho/Rock and TGF-β/NADPH Oxidase (NOX)-1 are the most conspicuous ones which stimulate the overproduction of collagen (9). Infiltration of inflammatory cells such as macrophages, mast cells, and lymphocytes are shown to be involved in progression of lung injury (8).

In addition to TGF-β, several studies have emphasized that interleukin (IL)- 4 and IL-13 play a key role in the development of radiation-induced inflammation and fibrosis (10). Although the complete downstream signaling pathways for these two genes are yet unknown, it seems that prolonged stimulation of hydrogen peroxide (H_2_O_2_) may be involved in this process. A study by Hassani et al. showed that two subfamilies of NADPH oxidase enzymes, dual oxidase 1 & 2 (DUOX1 & 2), are responsible for chronic H_2_O_2 _production following IL- 4 and IL-13 upregulation (11). In this study, we aimed to evaluate possible protective features of curcumin against upregulation of *DUOX1* and *DUOX2* expression after exposure to an acute high dose of gamma radiation. We also examined changes in the levels of IL-4 and IL-13 genes and proteins. To verify data, we further evaluated the histopathological changes. The protective effect of curcumin was evaluated for all aforementioned pro-fibrotic markers.

## Materials and methods

**Experimental design**

Twenty male Wistar rats (eight week- old,

200±20g) were purchased from School of Pharmacy, Tehran University of Medical Sciences, Tehran, Iran, while curcumin was purchased from Sigma–Aldrich Co, St. Louis, MO, USA. Rats were divided into four groups (5 rats in each group):1- Radiation only; 2- Curcumin only; 3- Radiation + Curcumin; 4- Control group without receiving any drug or radiation. Group 1 received 15 Gy gamma rays to the chest region. Group 3 received similar dose at the same time. Oral gavage of curcumin (150 mg/kg) was carried out for 4 and 6 consecutive days before and after irradiation, respectively. On the day of irradiation and 1 h before exposure to gamma rays, administration of curcumin to rats was the same dose prior to irradiation. Rats were anesthetized and sacrificed 67 days after irradiation. The lung tissues were removed and dissected into two parts. The left part was fixed in 10% neutral buffered formalin, and the remaining parts were frozen at -70 ˚C for real time PCR and ELISA analysis.

**Irradiation and treatment with curcumin**

Irradiation of the chest area was performed using a cobalt-60 beam unit with a source to skin distance (SSD) of 60 cm, and dose rate of 109 cGy/min. Ten min before irradiation, the rats received intraperitoneal injection of ketamine (60 mg/kg) and xylazine (20 mg/kg) as anesthestic. Curcumin was dissolved in 20% ethanol at a concentration of 30 mg/ml, and each rat received 1 ml (150 mg/kg).

**Real time PCR**

Total mRNA was extracted from lung samples using TRIzol reagent (GeneAll, South Korea), followed by cDNA synthesis using cDNA synthesis kit (GeneAll, South Korea). Phosphoglucomutase 1 (*PGM1*) primer was used as internal control. All primers were designed by Gene runner and then blast in NCBI. The sequences of primers were as follows; *IL4Ra1*: forward 5’GAGTGAGTGGAGT CCCAGCATC3’; reverse5’GCTGAAGTAACAG GTCAGGC3’; *IL13Ra2*: forward 5’TCGTGTTAG CGGATGGGGAT3’; reverse 5’GCCTGGAAGCC TGGATCTCTA3’; *DUOX1*: forward 5’AAGAAA GGAAGCATCAACACCC3’; reverse 5’ACCAGG GCAGTCAGGAAGAT3’; *DUOX2*: forward 5’AG TCTCATTCCTCACCCGGA3’; reverse 5’GTAAC ACACACGATGTGGCG3’; *PGM1*: forward 5’CA TGATTCTGGGCAAGCACG3’; reverse 5’GCCA GTTGGGGTCTCATACAAA3’. Real time PCR was performed using Corbett Real time PCR (USA).

**ELISA**

The levels of IL-4 and IL-13 cytokines were measured via ELISA kit (Rat IL-4 and IL-13 kits, Zelbio, Germany), based on the procedure of manufacturer.

**Histopathological evaluation**

Fixation of lung tissues in 10% formalin was followed by embedding them in paraffin and subsequently obtaining 5 μm sections. These sections were stained with hematoxylin and eosin (H and E) as well as Masson trichrome (MTC) staining methods. The lung sections were evaluated as blinded by a pathologist with the aid of a light microscope at 100x magnification for various histological endpoints such as inflammation, fibrosis, vascular and alveolar damage, and inflammatory cells infiltration.

**Statistical analysis**

The results of study were expressed as mean ± standard deviation (SD) for each group. All analyses were performed using SPSS software Version 16 (SPSS, Inc, Chicago, IL, USA). Besides the statistical analysis of ELISA results was carried out using One Way ANOVA test (post Hoc Tukey’s HD), with Mann-Whitney test being used to analyze the histopathological data. Moreover, Real time PCR results were analyzed by t test. P values less than 0.05 were considered to be significant.

## Results

**Cytokines levels**

The results of ELISA showed that exposure of lungs to radiation led to a significant increase in the IL-4 levels (762±21 pg/ml) compared to control group (422±71 pg/ml) (P< 0.01). However, for rats treated with curcumin before and after irradiation, the IL-4 level decreased significantly (529±118 pg/ml) (P<0.05), while treatment with curcumin alone did not have such effect (400±17 pg/ml).The results of IL-13 showed no significant changes in all groups.

**Fig. 1:**
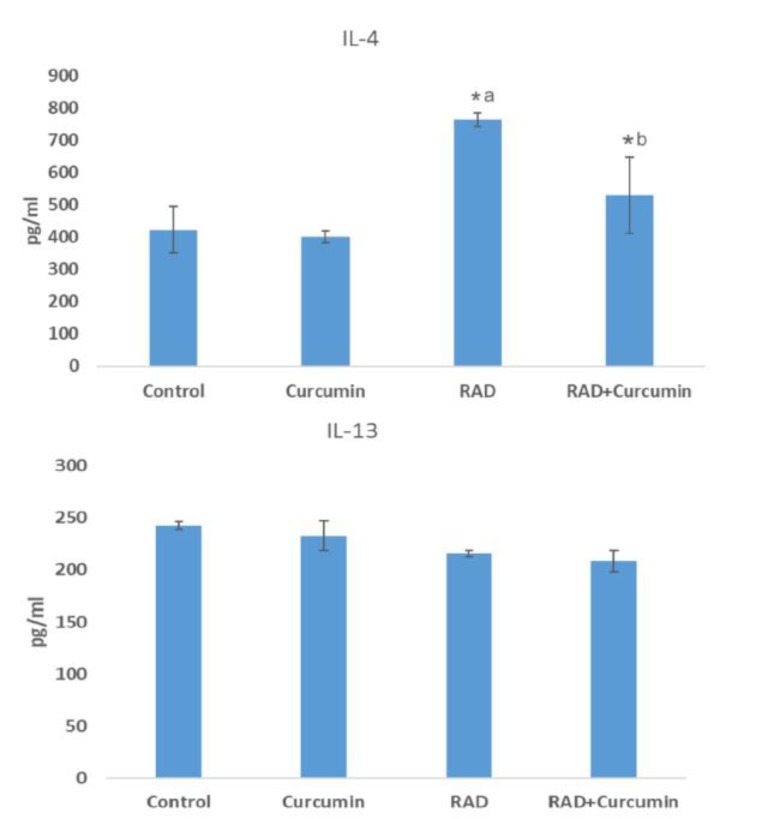
IL-4 and IL-13 cytokines levels following curcumin treatment, irradiation, and curcumin + irradiation. a: significant compared to control group; b: significant compared to irradiation group; Rad: irradiation. (ANOVA, P< 0.05)

**Fig. 2:**
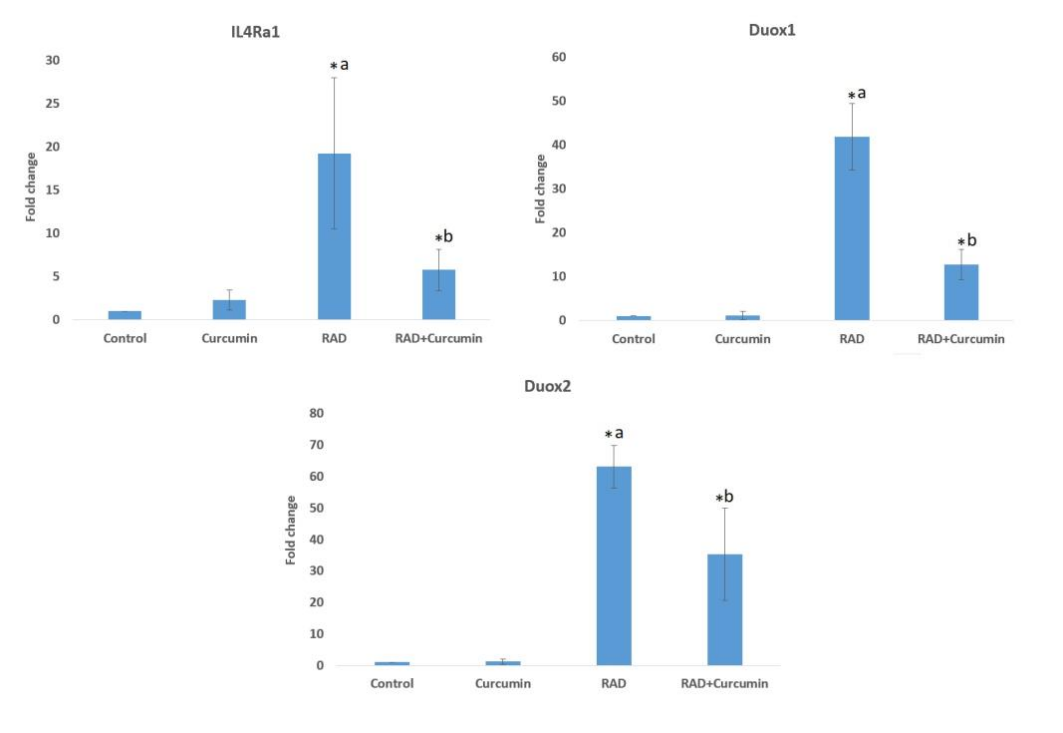
The expression of* IL4Ra1*, *DUOX1* and *DUOX2* following irradiation and curcumin treatment. a: significant compared to control; b: significant compared to irradiation group; Rad: irradiation; (T test, P<0.05)

**Table 1:** Histopathological results of curcumin treatment for alleviation of radiation induced lung injury

	**Control **	**Curcumin **	**Radiation **	**Radiation +Curcumin **
**Macrophage infiltration **	0±00	0±00	2.5±1.16^a^	0.83±1.26^b^
**Lymphocyte infiltration **	0±00	0±00	3±00^a^	1±00^b^
**Alveolar thickness **	0±00	0±00	1.66±1.15^a^	0±00^b^
**Vascular thickening **	0±00	0±00	1.00±00^a^	0±00^b^
**Edema **	0±00	0±00	1.00±1.00	0±00
**Fibrosis **	Absent	Absent	Mild	Absent

The evaluated factors were scored as 0=normal; 1= mild change; 2= severe; 3= more severe. Fibrosis was represented as absent, mild and severe.

a : significant compared to control;

b : significant compared to irradiation group.

**Fig. 3:**
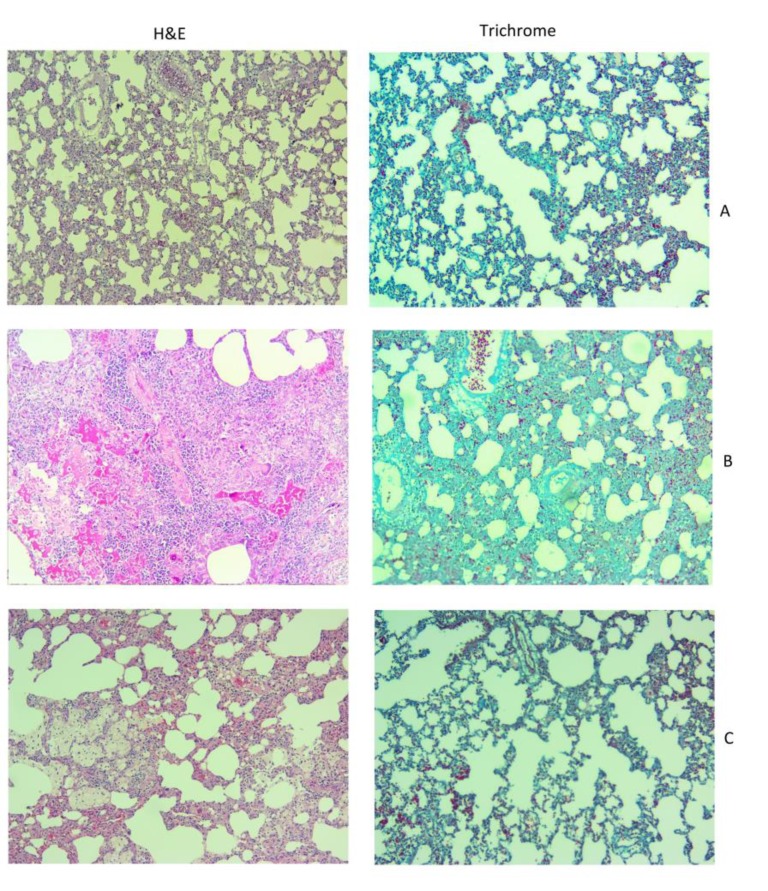
Histological image of radioprotective effect of curcumin on the lung tissue of rats. A: control; B: radiation; C: Radiation plus curcumin. In H&E images (×100) the severe infiltration of macrophages and lymphocytes are obvious, being attenuated following treatment with curcumin. Also, a mild fibrosis is seen in trichrome staining (×100) which ameliorated following administration of curcumin

**Gene expression analysis**

QPCR results showed that in gamma ray-irradiated rats, there was a remarkable increase in *IL4Ra1* expression (19.4±8.7 fold) (P<0.01). Treatment with curcumin significantly decreased the expression of *IL4Ra1* in comparison with the radiation group (5.7±2.4 fold) (P=0.001). None of the groups demonstrated the expression of *IL13Ra2* gene. Irradiation led to a meaningful increase in *DUOX1* expression (41.8±7.5 fold) in comparison with the control group (P=0.001). For the rats treated with curcumin before and after irradiation, the expression of *DUOX1* was reduced significantly (12.7±3.45 fold) (P<0.05). The results of *DUOX2* gene expression showed a significant increase following irradiation (63.1±6.7 fold), in comparison with the control group. Treatment with curcumin tremendously reduced this expression (35.3±14.6 fold) (P<0.05).

**Histopathological evaluation**

Histopathological results showed that exposure to ionizing radiation led to a noticeable infiltration of inflammatory cells and mild increase in vascular and alveolar thickening. In addition, mild fibrosis and inflammation were observed. All of these changes were shown to be relieved upon curcumin treatment (table 1).

## Discussion

Due to the increasing number of cancer patients who undergo radiotherapy, meticulous attention should be confined to the outcomes and side effects of this treatment modality. Knowledge on the molecular mechanisms of radiation injury in each organ can help make a decision about appropriate protocols to enhance therapeutic effects (12, 13). Exposure to ionizing radiation leads to massive free radical production and DNA damage, triggering cell death in irradiated organs within several hours or days. Cell death through apoptosis or necrosis results in the secretion of various inflammatory and anti-inflammatory cytokines as well as chemokines (14), probably followed by accumulation of macrophage, mast cells, lymphocytes, etc. The overproduction of various cytokines such as IL-1, IL-4, IL-6, IL-8, IL-13, IL-33, TNF-α, and TGF-β allows the inflammatory cells to generate ROS and NO. These changes are associated with continuous production of free radicals by ROS-NO-producing enzymes (15). This phenomenon is known as ROS induced ROS (16). Chronic oxidative damage stimulates collagen production which increases soft tissue stiffness. The most important ROS producing enzymes are cyclooxygenase-2 (COX-2), inducible nitric oxide synthase (iNOS), lipoxygenases (LOX), and NADPH oxidase subfamilies including NOX1-5 and DUOX1 & 2 (17). It comes with no surprise that the suppression of these enzymes can alleviate chronic effects of ionizing radiation after radiotherapy or following exposure to a high dose of radiation in a nuclear disaster (18).

In current study, we aimed to evaluate the expression of *DUOX1* and *DUOX2 *signaling pathways in the lung tissues after exposure to an acute high dose of gamma radiation. Moreover, we examined the protective effect of curcumin against upregulation of these pathways and the histopathological changes induced by radiation. Results showed no significant change in the level of IL-13 following irradiation or drug treatment. In addition, the expression of *IL13Ra2 *was not observed in the groups. Although not affecting the IL-13 levels, irradiation of lung tissues led to an 80% increase in the level of IL-4. The expression of *IL4Ra1* also increased by 19 fold. The results of *DUOX1 *&* 2* gene expression showed a significant expression for both of them, *DUOX1* expression by 41 fold, increase while the expression of *DUOX2* increased greater than 60 fold. These results clearly demonstrate that following exposure of lung tissues to radiation, the upregulation of the pro-oxidant enzymes may be involved in the late effects of radiation. Curcumin administration could reduce the level of IL-4 following irradiation. Furthermore, curcumin was able to potently suppress *IL4Ra1* and *DUOX2* expression. Although curcumin could decrease the expression of *DUOX1*, its suppressive effect on *IL-4*–*IL4Ra1*–*DUOX2* was shown to be stronger.

Histopathological results showed mild fibrosis, edema, vascular and alveolar thickening, accompanied by substantial infiltration of macrophages and lymphocytes. Curcumin could counter the increased fibrosis, edema, vascular and alveolar thickening. It was also able to attenuate macrophages as well as lymphocyte infiltration. As infiltration of macrophages and lymphocytes has been demonstrated to be directly associated with upregulation of IL-4 pathway, it seems that the curcumin-induced attenuation of IL-4 signaling pathways alleviates the late effects on lung tissues such as infiltration of inflammatory cells. In recent years, several studies have been conducted to evaluate radioprotective effect of herbal agents (19, 20). Curcumin as a natural radioprotective agent has shown interesting results for low toxicity and effective protection against oxidative damage and inflammatory responses (21-24). It can attenuate the expression of various inflammatory mediators such as transcription nuclear factor of κB (*NFκB*), cyclooxygenase 2 (*COX2*), inducible nitric oxide synthase (*iNOS*), lipoxygenases, inflammatory cytokines like *IL-1*, *IL-6*, *IL-8*, *TNF*, chemokines, and others (25-28). Moreover, curcumin has been shown to ameliorate radiation- induced fibrosis via inhibition of TGF-β pathways (29-31). Based on these promising results accompanied by low toxicity and sensitization of tumor cells, there is a growing interest to clinically utilize curcumin inradiotherapy.

Our findings also showed that treatment with curcumin can ameliorate late responses of lung tissue to ionizing radiation. Exposing lung tissues to 15 Gy gamma rays led to a significant upregulation of DOUX2 signaling pathway in this tissue. On the other hand, treatment with curcumin hindered this pathway. This study showed that irradiation of rat’s lung led to the upregulation of *IL-4 *and its downstream genes such as *IL4Ra1* and *DUOX2*. Although the expression of *DUOX1* increased following lung irradiation, it seems that its stimulation was independent of IL-13. Besides, upregulation of *IL-4* and its downstream genes was associated with infiltration of macrophages and lymphocytes, as well as increased histopathological damage. Curcumin could suppress IL-4–IL4Ra1–DUOX2 signaling and attenuate histopathological damages.
